# Shell Distribution of Vitamin K3 within Reinforced Electrospun Nanofibers for Improved Photo-Antibacterial Performance

**DOI:** 10.3390/ijms25179556

**Published:** 2024-09-03

**Authors:** Wenjian Gong, Meng-Long Wang, Yanan Liu, Deng-Guang Yu, Sim Wan Annie Bligh

**Affiliations:** 1 School of Materials and Chemistry, University of Shanghai for Science and Technology, Shanghai 200093, China; 223353279@st.usst.edu.cn (W.G.);; 2 School of Health Sciences, Saint Francis University, Hong Kong 999077, China

**Keywords:** personal protective equipment, coaxial electrospinning, core–shell, vitamin K3, photo-antibacterial

## Abstract

Personal protective equipment (PPE) has attracted more attention since the outbreak of the epidemic in 2019. Advanced nano techniques, such as electrospinning, can provide new routes for developing novel PPE. However, electrospun antibacterial PPE is not easily obtained. Fibers loaded with photosensitizers prepared using single-fluid electrospinning have a relatively low utilization rate due to the influence of embedding and their inadequate mechanical properties. For this study, monolithic nanofibers and core–shell nanofibers were prepared and compared. Monolithic F1 fibers comprising polyethylene oxide (PEO), poly(vinyl alcohol-co-ethylene) (PVA-co-PE), and the photo-antibacterial agent vitamin K3 (VK3) were created using a single-fluid blending process. Core–shell F2 nanofibers were prepared using coaxial electrospinning, in which the extensible material PEO was set as the core section, and a composite consisting of PEO, PVA-co-PE, and VK3 was set as the shell section. Both F1 and F2 fibers with the designed structural properties had an average diameter of approximately 1.0 μm, as determined using scanning electron microscopy and transmission electron microscopy. VK3 was amorphously dispersed within the polymeric matrices of F1 and F2 fibers in a compatible manner, as revealed using X-ray diffraction and Fourier transform infrared spectroscopy. Monolithic F1 fibers had a higher tensile strength of 2.917 ± 0.091 MPa, whereas the core–shell F2 fibers had a longer elongation with a break rate of 194.567 ± 0.091%. Photoreaction tests showed that, with their adjustment, core–shell F2 nanofibers could produce 0.222 μmol/L ·OH upon illumination. F2 fibers had slightly better antibacterial performance than F1 fibers, with inhibition zones of 1.361 ± 0.012 cm and 1.296 ± 0.022 cm for *E. coli* and *S. aureus*, respectively, but with less VK3. The intentional tailoring of the components and compositions of the core–shell nanostructures can improve the process–structure–performance relationship of electrospun nanofibers for potential sunlight-activated antibacterial PPE.

## 1. Introduction

With the sudden epidemic of the 2019 coronavirus disease (COVID-19), personal protective equipment (PPE) experienced explosive development, with various protective materials having been proposed [1,2]. Some commonly used PPE, including masks, protection suits, isolation gowns, surgical gowns, and goggles, can protect human beings from polluted liquid and/or air environments to reduce the risk of cross-infection from hazardous substances. Even though PPE has been developed for several decades, there is much room for improvement in its protective capability and comfort aspects. Especially during major public health events, high protective capability will save many lives [3,4,5,6,7]. Numerous publications in the Web of Science (https://webofscience.clarivate.cn/wos/alldb/basic-search) expresses the same idea. Searching “personal protective equipment” as a topic word revealed 3363 documents published between 2015 and 2019,while the number sharply increased to 15,685 between 2020 and 2024 (search date: 21 July 2024). It is conceivable that many existing and newly emerging bacteria and viruses (including the virus that caused COVID-19) will coexist with human beings for a long period [8]. New PPE materials with antibacterial properties, breathability, portability, and certain mechanical properties can be applied in various scenarios. Nanofiber membranes, as a form of nanomaterial, have been broadly explored in many fields for numerous applications, such as antibacterial use, drug release systems, and air filtration.

For now, wearing PPE is still an effective and robust way to protect people from infections with bacteria and viruses. Most PPE is made of synthetic fabric, which is breathable, has high mechanical strength, and can be efficiently produced. Diverse methods for the fabrication of membranes have been proposed, such as interfacial polymerization [9,10], secondary growth [11], chemical vapor deposition [12], vapor printing [13], melt extrusion [14,15], electrospinning [16,17], and electrospraying [18,19,20]. Compared with traditional fabric products, non-woven fabrics, such as electrospun fiber membranes, have smaller fiber diameters and denser arrangements, which will enable better resistance to bacteria and viruses. Electrospinning is a top-down nanofiber production technique, which is easier to scale up than bottom-up synthesis [21,22,23,24]. Under the action of an electric field, the polymer solution overcomes the surface tension and is deposited on the receiving aluminum foil due to the electrostatic force. With the continuous advancement of electrospinning technology, multi-fluid working forms, such as bi-fluid and tri-fluid electrospinning, have been derived from traditional monoaxial electrospinning, which uses only one working solution to prepare monolithic nanomaterials [25,26,27,28]. Electrospinning also produces a variety of structural forms, such as the most fundamental bi-chamber core–shell and Janus (or side-by-side), tri-chamber core–shell, tri-layer Janus, and multi-chamber combinations of core–shell and Janus structures [29,30,31]. They can be designed according to the needs of practical applications to improve the performance of polymers and the added active ingredients. In this research, coaxial electrospinning was used to prepare core–shell nanofibers that possess a certain degree of toughness and can be applied to PPE or air filtration [32,33,34].

With the continuous emergence of public health incidents in recent years, public awareness of personal protection has never been higher. Especially with the rampant outbreak of respiratory diseases, the demand for PPE among healthcare workers and the general public has increased. Due to increasing personal protection awareness and social requirements, almost everyone who goes out needs to wear corresponding protective equipment. PPE masks and protective clothing have become essential equipment in daily life. The mass purchasing and hoarding of PPE has also posed significant challenges to PPE production methods and output. Ordinary PPE can only serve as a barrier against bacteria and viruses and cannot inactivate them. Therefore, used PPE carrying a large number of viruses can cause secondary infections and other problems [35,36,37,38]. Photoactive substances that can produce reactive oxygen species (ROS) under light conditions are excellent green antibacterial agents and are widely used in fields such as biomedicine and food safety [39,40]. Vitamin K is a photoactive substance and has a powerful, instant inactivation effect on Gram-positive and Gram-negative bacteria under sunlight [41,42,43]. VK3 is also an essential element for blood coagulation and bone health and is a good additive material for photoinduced antibacterial PPE. In the field of micro-organisms, Yıldırım et al. thiolated VK3 to further enhance its antibacterial performance, which resulted in antibacterial activity with an MIC_50_ of 4.88 μg/mL against methicillin-resistant *Staphylococcus aureus* (MRSA) strains, thus this modification exhibits good application potential [44]. Chadar et al. prepared n-alkylamino derivatives of VK3 with minimum inhibitory concentrations against *Pseudomonas aeruginosa* and *S. aureus* of 0.625 μg/mL and 0.3125 μg/mL, respectively, and the mechanism of their antibacterial activity was investigated. As a result, a new method of VK3 modification was proposed and discussed [45].

Electrospinning differs from the traditional single-fluid blending process and can be implemented in coaxial, side-by-side, and complex multi-fluid processes [46,47,48,49,50]. Improvements in the mechanical properties of nanofibers using multi-fluid electrospinning have been reported. In general, to improve a weak polymer phase, a high-strength material is often incorporated. For example, Cai et al. used polyvinylidene fluoride (PVDF, 26.04 MPa in 230 °C) to improve a polyimide (2.63 MPa in 230 °C) phase using side-by-side electrospinning [51]. The obtained PVDF/polyimide Janus fibers had 15.49 MPa stress at 230 °C. After that, they used thermoplastic polyurethane (TPU) to further improve PI [52]. Liu et al. combined polyvinylpyrrolidone (PVP) and polyethylene oxide (PEO) to obtain PEO-PANI/PVP Janus fibers with improved toughness [53]. The PEO-PANI side gives Janus nanofibers better ductility (67%), while the PVP side provides the fibers with better strength (12.3 MPa), which also demonstrates that different electrospun structures can effectively combine the properties of different polymers to achieve the best application effect.

Poly(vinyl alcohol-co-ethylene) (PVA-co-PE), a crosslinked polymer with high strength and abundant free hydroxyls, has been used for air filtration and antibacterial applications in many studies [54,55,56,57,58]. For instance, Yi et al. realized high-performance aerosol filtration with a suspension-drying method using PVA-co-PE [59], and the efficiency of the resultant filtration was more than 97.5%. Liu et al. harnessed a suspension-coating process to fabricate a Ag-nanoparticle-decorated poly-ethyleneimine-grafted PVA-co-PE nanofibrous membrane [53]. The prepared membrane reached 99.99% inactivation effectiveness. Liang et al. [60] modified PVA-co-PE with dime-thylol-5,5-dimethylhydantoin (DMDMH) and prepared a new rechargeable N-halamine antimicrobial material that effectively inactivated bacteria in three minutes (more than 99.9999%). Moreover, the new material had a high filtration efficacy (more than 99.5%) and beneficial mechanical properties (tensile stress of up to 7.3 MPa). Zhang et al. blended PVA-co-PE and PAN with VK compounds to investigate their photoinduced antibacterial ability in nanofibers [61]. The results indicated that the nanofiber membrane with the addition of VK compounds produced strong antibacterial effects (>99.9%) in a short period of time (<90 min) and demonstrated that PVA-co-PE is a more suitable polymer material for such applications. However, given the low toughness of PVA-co-PE nanofibers prepared by electrospinning, polyethylene oxide (PEO) was used as the core to reinforce the resultant nanofibers by strengthening its weak links,. The prepared reinforced core–shell nanofibers possessed a PVA-co-PE shell loaded with VK3. As a naturally photoactive material, VK3 performs well as an antibacterial agent in a solar environment. This has been attributed to the ROS produced by the photoreaction of VK3 [62,63,64].

In this study, we conducted coaxial electrospinning to prepare core–shell nanofibers, in which the core and shell components and compositions were intentionally tailored for better mechanical properties and improved functional performance. A copolymer, PVA-co-PE, carrying vitamin K3 was used as the shell, and PEO was incorporated into the core of the core–shell nanofibers to improve their toughness. According to previously published works, VK3 possesses light-induced antibacterial properties when combined with PVA-co-PE [61]. The distribution of PVA-co-PE and VK3 only on the shell was anticipated to increase its usage efficiency.

## 2. Results and Discussion

### 2.1. Implementation of Coaxial Electrospinning

**Figure 1:**
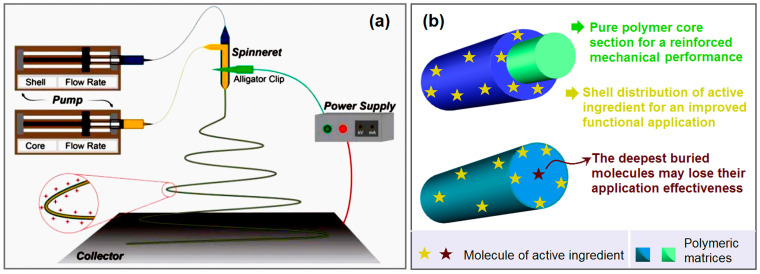
Coaxial electrospinning and core–shell nanofibers: (**a**) a diagram of the coaxial electrospinning system equipped with four primary components; (**b**) the design strategy for advanced functional nanomaterials based on core–shell nanofibers.

A diagram of the coaxial electrospinning process is shown in Figure 1a. The implementation of coaxial electrospinning is almost the same as single-fluid blending electrospinning. The only difference is that two syringe pumps are simultaneously switched on to drive both the core and shell working fluids to the electrical field through a concentric spinneret. However, the resultant core–shell nanofibers are able to provide a powerful platform for fabricating numerous advanced functional nanomaterials. In this study, the strategy has two key elements (Figure 1b): one is the pure PEO core section for the reinforced mechanical performance, and the other is the shell distribution of VK3 molecules for the antibacterial function, preventing the potential waste of VK3 embedded in the innermost section of monolithic composite nanofibers.

In this study, two electrospinnable working fluids were prepared. Fluid 1 consisted of 5% (*w*/*v*) PEO, 4% (*w*/*v*) PVA-co-PE, and 0.8% (*w*/*v*) VK3 in a solvent mixture of n-propyl alcohol and deionized water with a volume ratio of 7:3. Fluid 2 comprised 5% (*w*/*v*) PEO in a mixed solvent of n-propyl alcohol and deionized water with a volume ratio of 7:3. Two types of nanofibers were fabricated. Monolithic nanofibers (F1) were directly prepared from Fluid 1 (i.e., the shell working fluid) using a single-fluid blending electrospinning process. Core–shell nanofibers (F2) were prepared using coaxial electrospinning. The experimental parameters of the operations and the theoretical contents of VK3 within the F1 and F2 nanofibers are included in Table 1.

**Figure 2:**
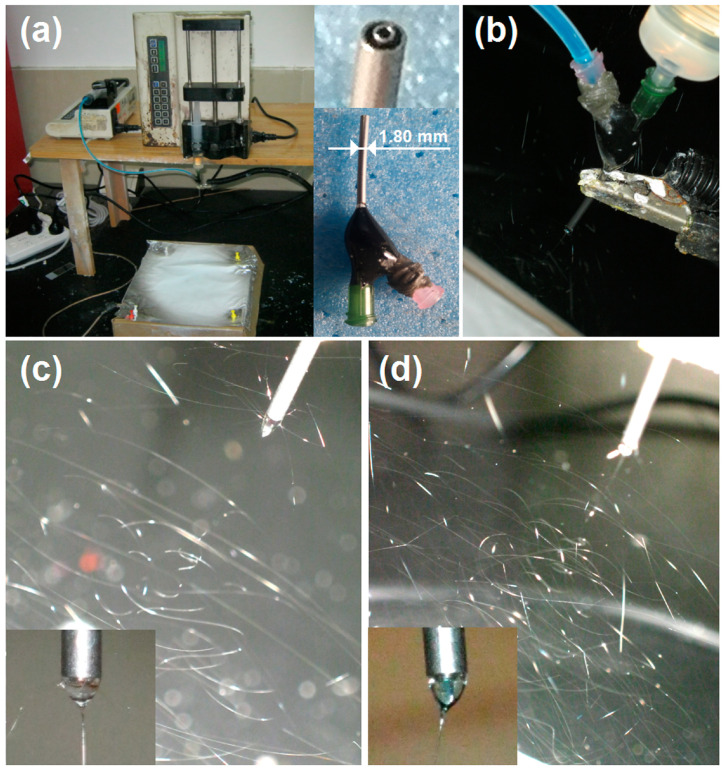
The implementation of single-fluid blending electrospinning and the coaxial electrospinning process: (**a**) a view of the whole homemade electrospinning system, where the top-right and bottom-right insets show the concentric spinneret’s outlet and its whole structure, respectively; (**b**) the connections of the concentric spinneret with the syringes and power supply; (**c**) typical single-fluid electrospinning of the shell solution, where the bottom inset shows a typical Taylor cone; (**d**) a typical coaxial electrospinning process, where the bottom inset shows a typical compound Taylor cone.

The declaration that electrospinning is a facile process for producing polymeric nanofibers has two implication. One is that the nanofibers are produced in a single step and a straightforward manner. The other is that the electrospinning system is easy to set up. The whole homemade electrospinning system, which was simply composed of a power supply, two syringe pumps, a spinneret, and a collector, is presented in Figure 2a. The spinneret is the key element [65,66,67]. In this study, a homemade concentric spinneret was utilized, which was composed of a G22 stainless steel capillary (with inner and outer diameters of 0.51 mm and 0.82 mm, respectively) for guiding the core fluid and a G15 stainless steel capillary (with inner and outer diameters of 1.36 mm and 1.80 mm, respectively) for directing the shell fluid. Its outlet and its whole structure are shown in the top-right and bottom-right insets of Figure 2a, respectively. Figure 2b shows a convergent point of a series of elements, including all of the working fluids and an alligator clip for transferring electrostatic energy from the power supply to the working fluids.

The initiation of any electrohydrodynamic atomization (EHDA, including electrospinning, electrospraying, and e-jet printing) requires sufficient electrostatic energy to overcome the surface tension of the liquid pumped out from the outlet of the spinneret [68,69]. If the working fluid is electrospinnable, it is an electrospinning process; otherwise, it is an electrospraying procedure [46,70]. In this study, both Fluid 1 and Fluid 2 had sufficient electrospinnability. Thus, the homogeneous F1 nanofibers could be fabricated robustly and continuously. A typical digital image of the single-fluid electrospinning of the shell solution is given in Figure 2c. The process includes three well-known steps, i.e., the formation of a Taylor cone, a straight fluid jet, and an unstable region with a gradual increase in unstable whipping and bending loops. When the core fluid was simultaneously pumped into the spinneret, the process became a typical coaxial electrospinning process. The shell and core fluids were highly compatible because they were prepared from the same solvent mixture of n-propyl alcohol and deionized water with a volume ratio of 7:3. As anticipated, the coaxial process is smooth and robust, as the digital photos in Figure 2c,d show.

### 2.2. Morphology and Inner Structure

**Figure 3:**
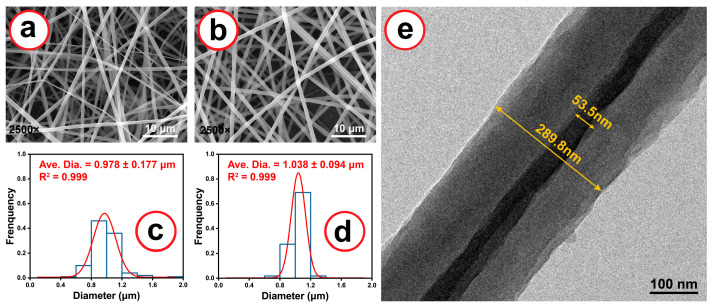
SEM images of prepared fibers: (**a**) F1 and (**b**) F2; diameter distribution of corresponding fibers: (**c**) F1 and (**d**) F2; (**e**) TEM image of F2.

Figure 3a–d show the SEM images and diameter distributions of the nanofibers. The blended electrospun nanofibers, F1, do not have a very uniform diameter size, with an average of 0.978 ± 0.177 μm, which might have been caused by some unstable moments during the uniaxial electrospinning process. In contrast, coaxial electrospun fibers have a much more uniform diameter distribution, with an average of 1.038 ± 0.094 μm. Compared with F1, F2 has a bigger average diameter, which could be due to the slightly higher total feed rate compared to that used for F1 at the same applied high voltage. Through coaxial electrospinning, F1 was coated on the linear PEO core surface. Thus, this technology can ensure a larger surface area. Moreover, the core–shell structure will generally grant composite fibers a high tensile strength.

The TEM image in Figure 3e shows that the fiber has a distinctive core–shell structure with a 289.8 nm diameter and an approximately 53.5 nm core diameter. This is consistent with the structure of the concentric spinneret. It is also clear that the surface of the nanofiber has numerous shallow sags and crests. The diameter is much smaller than the value obtained from the SEM image. This discrepancy could be caused by the process of sample preparation, which can result in the stretching of the nanofibers and the shrinkage of their diameters. Meanwhile, the core section is very slim, which may be attributable to the above-mentioned sampling and also the fact that the solute in the core fluid was only PEO, with a concentration of 5% (*w*/*v*). In contrast, the solutes in the shell fluid included PEO, PVA-co-PE, and VK3 at a higher overall concentration of 9.8% (*w*/*v*), almost twice that of PEO in the core fluid.

### 2.3. Physical State and Compatibility

**Figure 4:**
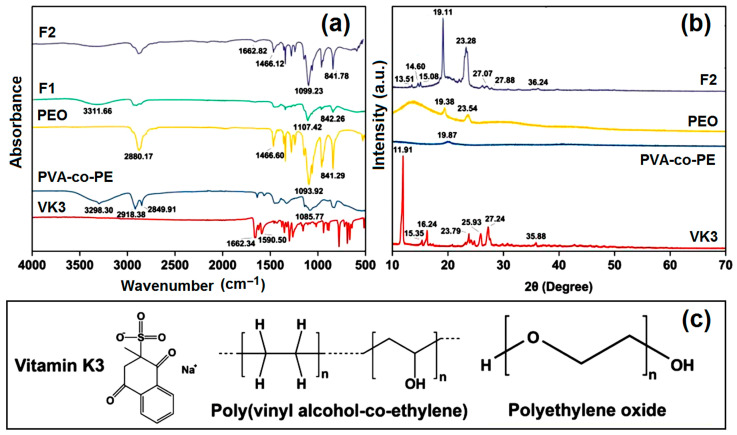
The physical state and compatibility of materials: (**a**) FTIR spectrograms and (**b**) XRD profiles of raw materials and prepared nanofibers; (**c**) structural formulas of VK3, PVA-co-PE, and PEO.

The FTIR spectra, XRD patterns, and molecular formulas of the raw materials are included in Figure 4. Figure 4a shows that the FTIR curve of the PVA-co-PE fiber membrane shows four main absorbance peaks: 3298 cm^−1^ due to O-H stretching, 2917 cm^−1^ and 2849 cm^−1^ due to C-H bending in CH_3_ and CH_2_, and 1085 cm^−1^ due to C-O stretching [71]. In the PEO curve, the peaks at 1093 cm^−1^ and 841 cm^−1^ indicate the asymmetric stretching and bending of C-O-C, respectively [72]. The two nanofibers form O-H and C-O-C bonds due to PVA-co-PE and PEO, respectively. As for VK3, the characteristic peak at 1662 cm^−1^ is attributed to C=O vibration. The C-C stretching vibration of an aromatic group is responsible for the peak at 1590 cm^−1^, which can be observed in the F1 and F2 curves as well. As indicated by its molecular formula, VK3 has a benzene ring and C=O and S=O groups; thus, it is easy for VK3 molecules to form hydrogen bonds with -OH within the molecules of PVA-co-PE and PEO. Meanwhile, its benzene ring can form large π electron clouds with the long carbon chains of two polymeric matrices.

X-ray diffraction (XRD) patterns present the physical states of components within the polymeric composites [73,74,75,76]. In Figure 4b, the XRD data show that raw VK3 is a typical crystal with some sharp peaks, and PEO has a degree of crystallinity because its XRD pattern has two peaks at 19.38° and 23.54°, which also appear at similar positions in the XRD pattern of the F2 nanofiber. In F2 nanofibers, VK3 can recrystallize during the solidification period. Some small peaks in the F2 XRD pattern can be attributed to VK3, such as 15.08°, 27.07°, 27.88°, and 36.24°. As shown in the SEM image, VK3 was not severely agglomerated. Using the large surface area of electrospun nanofibers, the utilization of VK3 can be drastically improved.

### 2.4. Mechanical Properties

**Figure 5:**
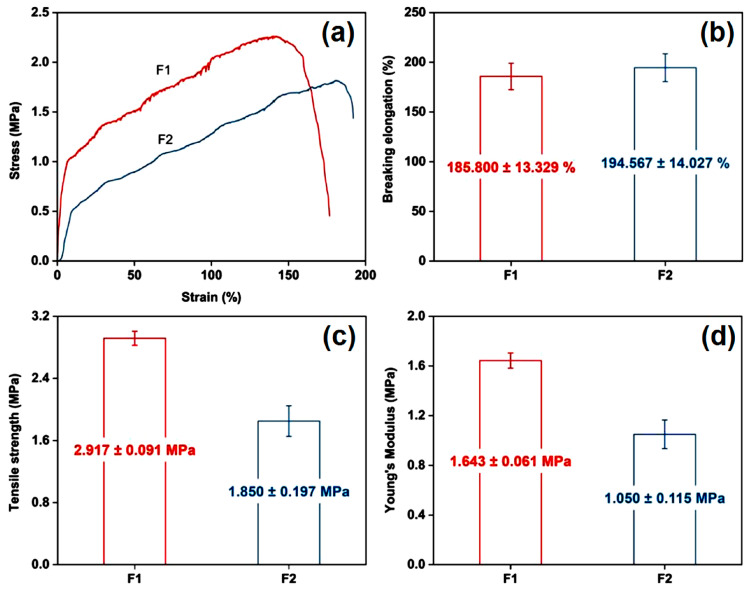
Analysis of material mechanical properties: (**a**) micro tension tests on fibrous membranes and some key properties: (**b**) breaking elongation, (**c**) tensile strength, and (**d**) Young’s modulus.

Reinforcement can be inferred from micro tension tests. Three nanofiber membranes were applied in these tests, and the curves (stress versus strain) are shown in Figure 5. Both prepared non-woven membranes display good toughness and strength. They both have more than 150% strain, and the breaking elongation is 185.800 ± 13.329% for F1 and 194.567 ± 14.027% for F2. The higher elongation of F2 could be caused by the PEO core, which is a high-toughness material. It is clear that the two polymers have slightly different intrinsic mechanical properties. PEO can be used to extend the strain length, but PVA-co-PE is preferable for improving the tensile strength. However, the high content of PVA-co-PE in F1 grants it high tensile strength. F1 has better tensile strength (2.917 ± 0.091 MPa) than F2 (1.850 ± 0.197 MPa). Young’s modulus, which indicates the ability of the reacting material to resist elastic deformation, is lower for F2 (1.050 ± 0.115 MPa) than for F1 (1.643 ± 0.061 MPa), which demonstrates the toughness of F2. Mechanical properties are important performance indicators of PPE materials. Only PPE with high strength and toughness have substantial application value. Specifically, high strength can better protect the wearer, and high toughness can better adapt to the wearer’s various activities, reducing the inconvenience caused by wearing PPE.

### 2.5. Water Contact Angle Tests

**Figure 6:**
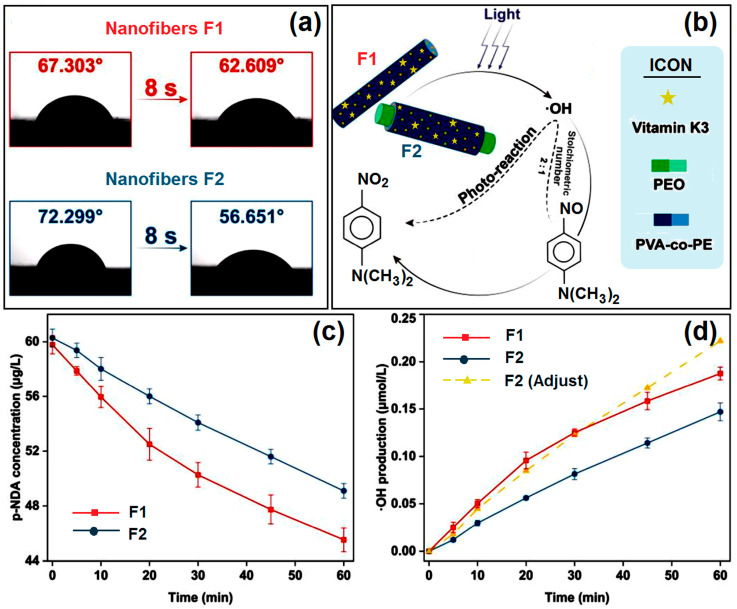
Water contact angle test and degradation test results for materials: (**a**) water contact angles of F1 and F2 at different times; (**b**) illustration of the degradation process; (**c**) degradation of p-NDA with different fibrous membranes; (**d**) ·OH production in 60 min.

Hydrophobicity was assessed using water contact angle tests. For this PPE membrane, good hydrophilic performance will promote the production of ROS, which can be generated by VK3 when exposed to an aqueous environment and illumination. Various body fluids can provide a natural aqueous environment for PPE. Thus, the hydrophilic performance of the prepared material will decide how well the environment can be utilized. Because PEO is a hydrophilic material, the prepared F1 and F2 nanofibers have high hydrophilicity. As shown in Figure 6a, they both have low WCAs: 67.303 ± 0.872° (F1) and 72.299 ± 1.922° (F2). Having many hydroxyl groups, on the one hand, can promote the production of ·OH and, on the other hand, can enhance the hydrophilic performance.

### 2.6. Photoreaction Tests

A photoreaction was conducted to degrade p-NDA, which reacts with ·OH in a certain ratio: two hydroxyl radicals can reduce one p-NDA molecule (shown in Figure 6b). In Figure 6c, it can be easily observed that the concentration of p-NDA continuously decreased under a xenon lamp for 60 min. F1 and F2 degraded 23.857 ± 0.917% and 18.530 ± 1.043% of p-NDA, respectively. According to the stoichiometric relationship between ·OH and p-NDA, we demonstrate the production of ·OH as well. However, in these tests, we used fibrous membranes with the same weight, whereas the mass percentages of VK3 in uniaxial and core–shell fibers were different. Thus, using an adjustment process, we found that ·OH production using F2 reached 0.222 μmol/L in 60 min, which is a little higher than when using F1 (0.188 ± 0.007 μmol/L), as illustrated in Figure 6d.

It can be seen that VK3 dispersed on the shell of the coaxial fiber has a higher utilization rate than VK3 dispersed in the monolithic fiber. As this photoreaction is most likely to occur on the surface of the nanofiber, the core–shell structure will help improve the utilization efficiency of VK3. Using the photoreaction test, the hydroxyl radicals generated with the different structures, F1 and F2, were quantified, which indicated that the nanofibers with the coaxial structure are more suitable for the photoreaction application of VK3 in the drug-loading mode. The generation of free radicals via photoinitiation is an important antibacterial method suitable for daily life. This experiment confirmed that adding photoactive VK3 to nanofibers is a new method that can be applied to PPE.

### 2.7. Antibacterial Tests

**Figure 7:**
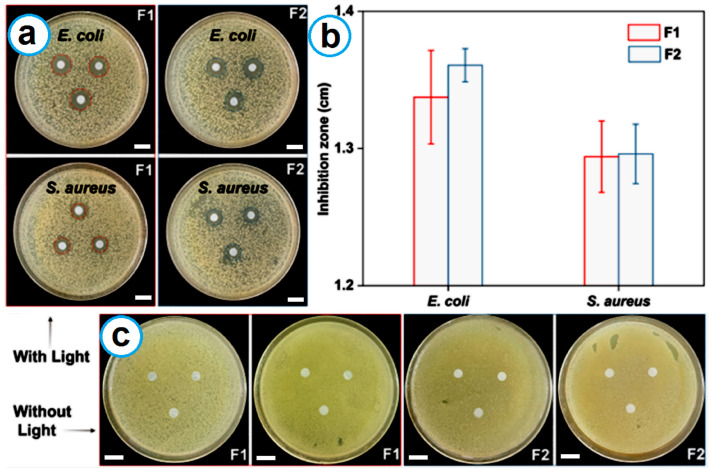
The photo-antibacterial performance of F1 and F2: (**a**) inhibition zones under light; (**b**) a histogram of inhibition zones; (**c**) inhibition zones without light. The scale bar represents 1 micron.

The antibacterial performance of the two prepared fibrous membranes under illumination was evaluated in a 37 °C incubator. The experimental results are included in Figure 7a–c. After 24 h of cultivation under light at a constant temperature, both F1 and F2 showed clear inhibition zones in both *S. aureus* and *E. coli*, indicating that both materials have good antibacterial activity under light. F1 produced 1.337 ± 0.034 cm and 1.294 ± 0.026 cm inhibition zones for *E. coli* and *S. aureus*, respectively. F2 has slightly higher antibacterial performance than F1: 1.361 ± 0.012 cm and 1.296 ± 0.022 cm for *E. coli* and *S. aureus*, respectively. Although these data are not statistically significant, the VK3 content in F2 was 5.41% (*w*/*w*), whereas that in F1 was 8.16% (*w*/*w*). With only 66% of the content in F1, the core–shell F2 nanofibers were still able to generate slightly larger zones, indicating better antibacterial performance. The antibacterial performance can certainly be further improved using two approaches in the future. One approach is to increase the flow rate of the core fluid or decrease the flow rate of the shell fluid to create core–shell nanofibers with a thinner shell section. The other is to adjust the VK3 concentration in the working fluid.

For comparison, without light irradiation, neither sample produced inhibition zones, which further reflects the photoinduced antibacterial activity of VK3. It is precisely because of the existence of VK3 that F1 and F2 can produce ROS such as ·OH. These ROS have the ability to eliminate bacteria. Therefore, the two prepared fibrous membranes have antibacterial activity under sunlight conditions and can function as PPE or a part of PPE. The results reported here suggest that polymers, regardless of whether they have antibacterial effects [77,78,79] or are inert as matrices [68,80,81,82], can be facilely converted using electrospinning into certain forms for a wide variety of functional applications, including PPE.

The development of bioactive materials for specific functional applications involves a series of methods [83,84] and mainly relies on two aspects, i.e., the reasonable selections of raw ingredients and material conversion technologies [85,86,87]. In this nano era, nanotechnologies are frequently introduced into the biomedical and healthcare fields [88,89,90,91]. The popularity of electrospinning and the resultant biomedical nanofibers are fine demonstrations [92,93,94,95]. However, as nanoscience and nanoengineering progress to “small, order, structure, and device”, multiple-fluid electrospinning and the related multi-chamber nanostructures will play an increasingly important role in realizing elaborate material designs for functional applications [34,96,97]. This investigation is just an example of developing advanced bioactive core–shell nanomaterials with an improved functional performance resulting from the shell distribution of the active ingredient and a pure core polymeric matrix. In the literature, numerous nanofiber-based bioactive nanomaterials are reported in which the active ingredients are homogeneously distributed throughout the polymeric matrices. Based on the protocols reported here, all such medical nanofibers can be updated to improve their functional performance simply by concentrating the active ingredient on the surface. In addition, some recent publications have revealed a series of strategies for developing novel PPE [98,99,100], and an effective combination of those strategies and the present core–shell VK3-loaded nanofibers is expected to bring about new types of nano PPE in the future.

## 3. Materials and Methods

### 3.1. Materials

Polyethylene oxide (PEO, M_W_ = 300,000) and menadione (also named vitamin K3, VK3, 98%) were purchased from Shanghai Macklin Biochemical Co., Ltd. (Shanghai, China). Poly(vinyl alcohol-co-ethylene) (PVA-co-PE, 32% ethylene) was obtained from Aldrich Chemical Co., Inc. (Shanghai, China). Propanol (AR, 99.5%) and PBS tablets were provided by Sinopharm Chemical Reagent Co., Ltd. (Shanghai, China). N,N-Dimethyl-4-nitrosoaniline (p-NDA, 98%) was obtained from Shanghai Macklin Biochemical Co., Ltd. (Shanghai, China). Other solvents and reagents were analytically pure. Deionized water was used in all preparations.

### 3.2. Preparing Nanofibers Using Coaxial Electrospinning

For coaxial electrospinning, Table 1 shows the methods used for core and shell solution preparation. In the two electrospinning processes, the collector distance was fixed at 17 cm. The flow rate ratio of core versus shell working fluids was controlled at 1:1 in volume, and the applied voltage was altered to the appropriate value listed in Table 1. On the basis of these parameters, nanofibers can be prepared continuously and stably.

In the homemade coaxial electrospinning apparatus, the two working fluids were driven by two pumps (KDS100, Cole-Parmer, Holliston, MA, USA) into the core and shell of the spinneret, separately. The working fluids gathered at the tip of the spinneret, and under the high-voltage electrostatic field provided by the power supply (ZGF 60 kV/2 mA, Wuhan Hua-Tian Corp., Wuhan, China), a Taylor cone gradually formed. When the electrostatic repulsion overcame the viscosity force within the Taylor cone, the solution was injected, and two stages were noted: a straight jet and whipping and swinging. The working fluid was solidified and then collected using the aluminum foil collector (Figure 2a).

### 3.3. Characterizations

The morphology and inner structure of the obtained nanofibers were observed by using field emission scanning electron microscopy (SEM, FEI Quanta450FEG, FEI Corporation, Hillsboro, OR, USA) and transmission electron microscopy (TEM, JEM 2200F, JEOL, Tokyo, Japan), respectively. For SEM, samples were observed at 20 kV after they had been gold-sputtered with a 5 nm thick coat using a vacuum turbo evaporator (Q150TES, Quorum, UK). In TEM tests, nanofiber samples were displayed on copper grids and observed at a high voltage (100 kV). The diameters of the fibers in the SEM and TEM images were measured using ImageJ V1.8.0 (National Institutes of Health, Bethesda, MD, USA).

The physical state and chemical compatibility of the nanofibers were determined using X-ray diffraction (XRD, D8 ADVANCE, Bruker, Karlsruhu, Germany) and Fourier transform infrared spectroscopy (FTIR, Spectrum 100, Perkin-Elmer, Billerica, MA, USA). In XRD analysis, samples were analyzed at a voltage of 40 kV and a current of 40 mA. Data were collected over a 2θ range from 10° to 70° at 5° per minute. In FTIR tests, data were collected in the range from 4000 to 450 cm^−1^ at a resolution of 2 cm^−1^, and each sample was scanned eight times.

The water contact angles (WCAs) of different samples were determined with an instrument to measure interfacial tension (JC2000C1, Shanghai Zhongchen Digital Technology Apparatus Co., Ltd., Shanghai, China). For this measurement, deionized water was used as the test liquid. Samples with a 5 cm length and a 2 cm width were used for water contact angle testing. One droplet of water was dropped on the sample, and the wetting process was recorded using a built-in high-speed camera. The corresponding results were measured using ImageJ with six repetitions and recorded as mean ± S.D.

Micro-tensile tests were implemented using rectangular samples from different prepared nanofiber membranes. The geometrical parameters of the different samples were kept at 50 mm in length and 20 mm in width. Their thickness was measured with a micrometer caliper, and the thickness values were 0.107 mm for F1 and 0.104 mm for F2. A micro-tensile testing machine (ZwickRoell GmbH & Co. KG, Ulm, Germany) was used in this test, and the distance between clamps was fixed at 20 mm. The model selected was fiberboard.

**Figure 8:**
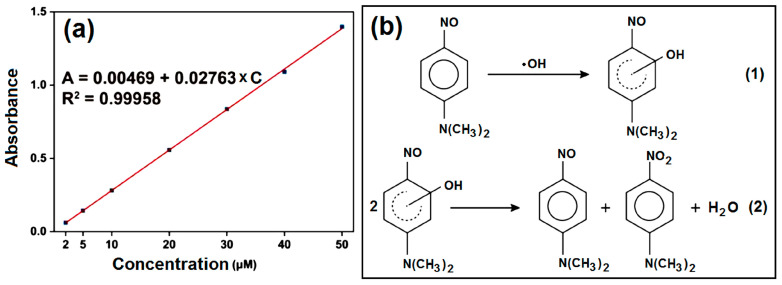
The standard curve and photoreaction process of p-NDA: (**a**) the standard curve of p-NDA; (**b**) the photoreaction that degrades p-NDA.

Photoreaction tests were carried out using different nanofiber membranes. A 50-milligram membrane was immersed in 50 mL of 50 μM p-NDA and put under a xenon lamp to be irradiated for different times (0, 5, 10, 20, 30, 45, and 60 min). The distance between the modulator tube and the samples was fixed at 15 cm. The absorbance of the samples was tested using a UV–vis spectrometer (UV-2102PC, Unico Instrument Co., Ltd., Shanghai, China) at 440 nm. The standard curve of p-NDA was plotted in advance, and the linear equation is A = 0.00469 + 0.02763 × C with R^2^ = 0.99958. The amount of ·OH was calculated from the relationship between p-NDA and ·OH as reactants [101], as shown in Figure 8. Experiments were repeated three times, and the results were recorded as mean ± S.D.

The Kirby–Bauer disc diffusion method was used to evaluate the antibacterial performance of different samples. *Staphylococcus aureus* (*S. aureus*) and *Escherichia coli* (*E. coli*) were used to conduct these experiments. First, 300 μL of each bacterial solution (105 CFU/mL) was uniformly coated on a 10 cm Luria–Bertani nutrient agar plate. The nanofiber membrane samples were prepared as 6 mm circular plates and sterilized using ultraviolet irradiation for 30 min. Three samples from each group were placed on LB culture plates. The plates were put into an incubator for 24 h at 37 °C and exposed to light. All operations were performed in a sterile environment. After that, the inhibition zones were measured and recorded as mean ± S.D.

### 3.4. Statistical Analysis

The data are expressed as the mean ± standard deviation (S.D.). The statistical analysis of the data was performed using the analysis of variance (ANOVA). ANOVA was followed by the Dunnett test whenever necessary (Origin 8.1, Origin Lab, Northampton, MA, USA), and a value of *p* < 0.05 was considered statistically significant.

## 4. Conclusions

In this work, a blend of electrospinning and coaxial electrospinning was successfully implemented, and uniaxial fibers and core–shell fibers were prepared using different processes. SEM and TEM images indicated that the core–shell fibers had a more uniform size distribution and a clear core–shell inner structure. XRD and FTIR showed that the components had sufficient compatibility and that VK3 could be uniformly dispersed in monolithic fibers (F1) and the shell sections of core–shell nanofibers (F2). The two prepared fibrous membranes were both hydrophilic materials with acceptable mechanical performance. The photoreaction experiments showed that they both had the desired performance in degrading p-NDA. Compared with the monolithic F1 nanofibers from the single-fluid blending electrospinning, on one hand, the core–shell F2 nanofibers could ensure better mechanical performance through the pure core section. On the other hand, distributing VK3 only on the shell in F2 is conducive to a higher utilization rate of the loaded VK3 molecules. Of course, the core–shell nanofibers are more complex to fabricate, particularly on a large scale. Overcoming this challenge and exploring the many potential interesting applications of electrospun core–shell nanofibers are major objectives for their industrial production.

## Figures and Tables

**Table 1:** Experimental scheme of different electrospinning processes.

No.	Electrospinning	Core	Shell	Core/Shell Flow Rate (mL/h)	Voltage (kV)	Collector Distance (cm)	VE3 Content (%)
F1	Monoaxial	--	Fluid 1 ^a^	0.7	5.0	17	8.16%
F2	Coaxial	Fluid 2 ^b^	Fluid 1	0.5/0.5	5.0		5.41%

^a^ Fluid 1 consisted of 5% (*w*/*v*) PEO, 4% (*w*/*v*) PVA-co-PE, and 0.8% (*w*/*v*) VK3 in a solvent mixture of n-propyl alcohol and deionized water with a volume ratio of 7:3. ^b^ Fluid 2 comprised 5% (*w*/*v*) PEO in a mixed solvent of n-propyl alcohol and deionized water with a volume ratio of 7:3.

## Data Availability

The data supporting the findings in this manuscript are available from the corresponding authors upon reasonable.
